# Supplementary material for: Lattice reconstruction induced multiple ultra-flat bands in twisted bilayer WSe2
Source: Nat Commun. 2021 Sep 23;12:5601. doi: 10.1038/s41467-021-25924-6 (PMC8460827; doi:10.1038/s41467-021-25924-6)
Supplement: Supplementary file 1 — Supplementary Info [file 41467_2021_25924_MOESM1_ESM.pdf]

Supplementary information for

Lattice reconstruction induced multiple ultra-flat bands in twisted bilayer WSe_2

En Li[†], Jin-Xin Hu[†], Xuemeng Feng[†], Zishu Zhou, Liheng An, Kam Tuen Law*, Ning Wang*, Nian Lin*

Department of Physics, The Hong Kong University of Science and Technology, Hong Kong SAR, China

†These authors contributed equally to this work

*Correspondence to: phlaw@ust.hk; phwang@ust.hk; phnlin@ust.hk

Supplementary Note 1: Large-range STS measurement of the moiré site-dependent electronic structure

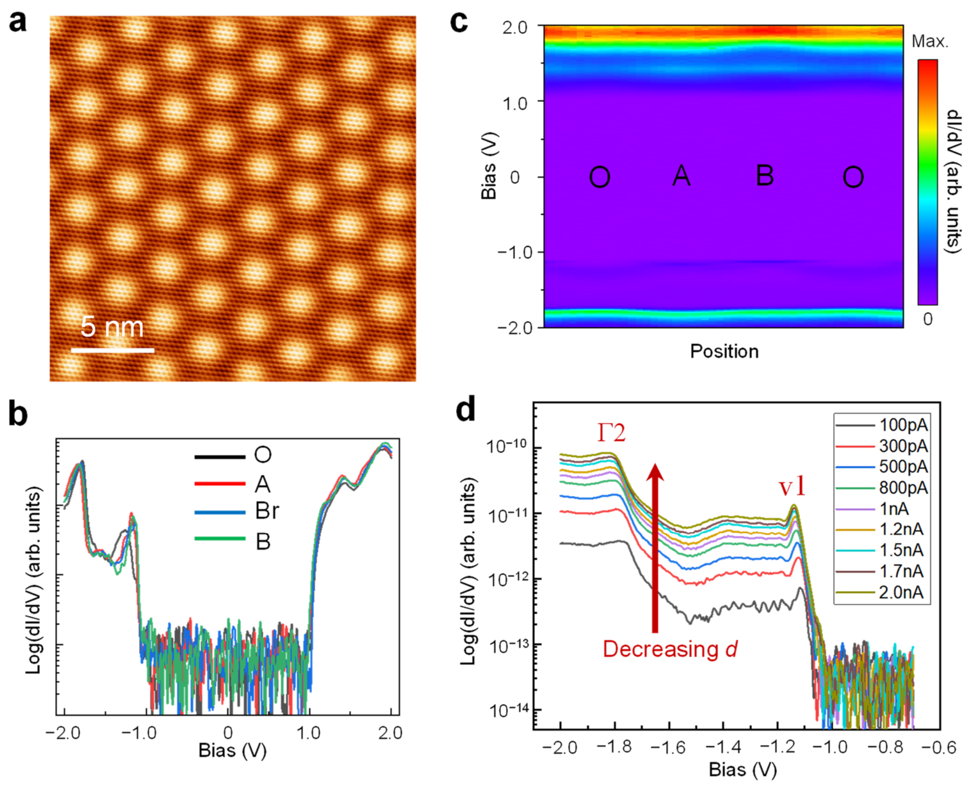

Supplementary Figure 1. Large-range STS measurement on the 54.1° TB-WSe_2. (a) An STM topographic image (−2.0 V, 1.0 nA) of 54.1° TB-WSe_2. (b) STS (V=−2.0 V, I=500 pA, V_{mod}=15mV) acquired at the center of four moiré sites, showing an energy bandgap and states in both the conduction and valence band. (c) Conductance map taken across O-A-Br-B-O sites, showing the moiré dependent states near valence band edges. (d) Tip–sample distance (*d*)-dependent STS measurement at the B site (V=−2.0 V, I= 100pA, 300pA, 500pA, 800pA, 1.0nA, 1.2nA, 1.5nA, 1.7nA and 2.0nA).

Supplementary Fig. 1a is an STM topographic image in twisted region of sample, showing a uniform moiré superlattice with a measured period of 3.2nm. The additional moiré pattern (around 1nm periodicity) caused by mismatch of WSe_2 and graphite lattice has not been observed in all the twist samples, indicating the graphite substrate plays a negligible role in reconstruction of bilayer WSe_2. On the different moiré sites, the measured logarithm of dI/dV spectra (Supplementary Fig. 1b) consistently show a

nearly intrinsic semiconductor bandgap (valence band maximum (VBM): −1.05 V, conduction band minimum (CBM): +0.99V), indicating a rather weak substrate doping effect. Together with previous ARPES analysis for TMD layers on graphite[1, 2], the influence of substrate on both reconstruction and flat-band formation can therefore be neglected.

Moreover, Supplementary Fig. 1d displays the height-dependent dI/dV spectra measured at site B in 54.1° sample. Previous studies showed that the STS peaks originated from K-point states in TMDs decay faster with decreased tip–sample distance (*d*)[3, 4]. In contrast, as shown in Supplementary Fig. 1d, the v1 peaks in our data show almost no decay, same behavior as the peak around −1.8V. Since the peak around −1.8V is from Γ point of WSe_2[5], the same height-dependence behavior evidences that the v1 peak corresponds to electronic states at Γ point. In addition, the slight energy shift of v1 and Γ2 peaks can be attributed to tip-induced band bending (TIBB) effect, due to poor screening of electric fields at such semiconducting surface[6]. As tip-sample separation decreasing, both v1 and Γ2 peaks in Supplementary Fig 1d shift toward deeper energy, agreeing well with the tendency of Γ-point states reported $MoSe_2$ monolayer[4] and WSe_2/WS_2 heterobilayer[3].

Supplementary Fig. 2 shows the large-range dI/dV spectrums taken across O-A-Br-B-O sites, with a twist angle ranging from 54.1° to 58.4°. The measured spatially dependent LDOS shows a good consistency at the varied twist angles, either for the flat bands or the states away from band edges. For example, the peaks at ~ −1.8 V and 1.3~2.0V show an energy downward shift at A sites and this downward shift increases as the twist angle approaches 60°. In contrast, this effect is almost negligible in 54.1° TB-WSe_2. This evolution of electronic structures with twist angles reveals the enhanced interaction effects between bilayer WSe_2, as the lattice reconstruction gradually involves in moiré structure.

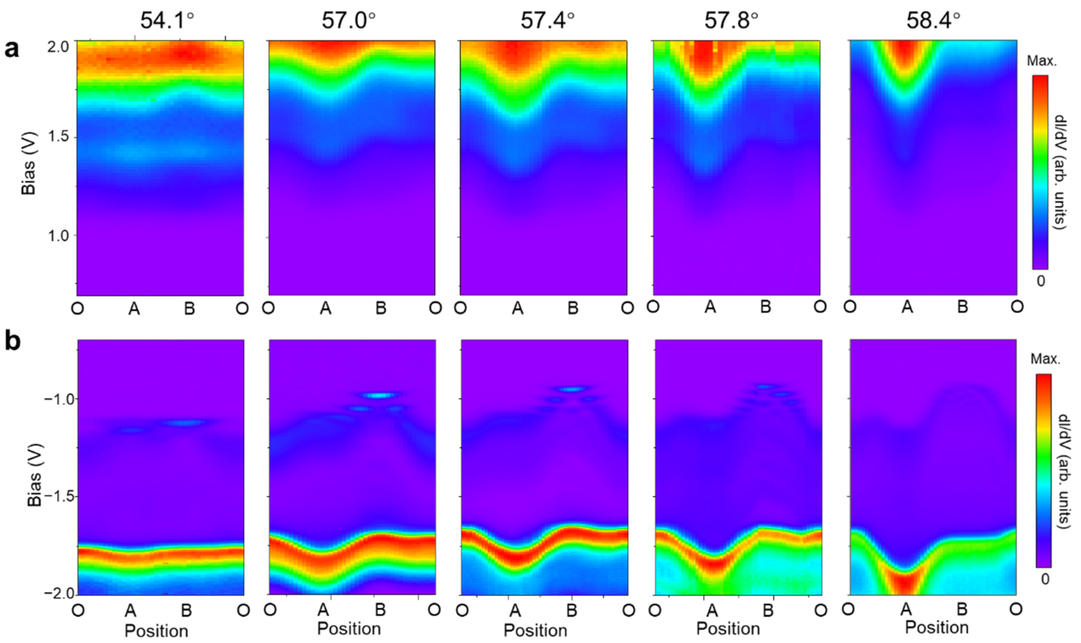

Supplementary Figure 2. Spatially dependent spectroscopy of 54.1°, 57°, 57.4°, 57.8° and 58.4° TB-WSe$_2$. (a) Conductance map taken along the O-A-B-O path at a bias voltage of 2.0V ~ 0.7V. (b) Conductance map taken along the O-A-B-O path at a bias voltage of −0.7V ~ −2.0V.

Supplementary Note 2: Estimation of the intrinsic bandwidth from dI/dV spectrum

We fit the obtained dI/dV spectrums by the theoretical formula in the following:

$$\frac{dI}{dV} = -\int_{-\infty}^{\infty}[\int_{-\infty}^{\infty}\rho(E)\,f'(\epsilon+E)dE]b(eV-\epsilon)d\epsilon \qquad \text{Supplementary Eq. (1)}$$

This equation represents the sample density of states $\rho(E)$ convolved with the thermal broadening $f'(\epsilon)$ and lock-in broadening b(V)[7]. Here we assume $\rho(E) = N(0,\sigma)$ is a Gaussian-like function with σ is the bandwidth, and

$$f'(\epsilon) = -\frac{\beta\exp(\beta\epsilon)}{[\exp(\beta\epsilon)+1]^2}, \beta = (k_B T)^{-1} \qquad \text{Supplementary Eq. (2)}$$

$$b(V) = \begin{cases} \frac{2}{\pi V_{mod}}\sqrt{1-\left(\frac{V}{V_{mod}}\right)^2}, & (|V| < V_{mod}) \\ 0, & (|V| > V_{mod}) \end{cases} \qquad \text{Supplementary Eq. (3)}$$

Based on the measured temperature of 5.3K and the modulation voltage (10 mV for 54.1°-57.8°, and 5 mV for 58.4°), we get the intrinsic bandwidths of the first B-confined peak with the twist angles. As labelled in Supplementary Table.1, the bandwidth decreases as the twist angle approaches 60°. In particular, at twist angles ranging from

57° to 58.4°, bandwidths decrease to be around a few meV only, acting as ultra-flat bands.

Supplementary Table1. The Gaussian fitted FWHM of the first band-edge peaks and corresponding deconvoluted intrinsic bandwidths at different twist angles

θ	FWHM (meV)	Intrinsic bandwidth(meV)
54.1°	37	15
57°	27	10.2
57.4°	24	8.8
57.8°	21.8	7.5
58.4°	15	5.8

We further estimate the intrinsic v1/v2 peak in 54.1° sample, to check whether v1 is an isolated flat band as shown in calculated band structure. The Gaussian fitted FWHM is ~37mV for v1 peak in site B, and ~55mV for v2 peak in site A. Based on the theoretical formula, the deconvoluted intrinsic bandwidth will be ~15mV for v1 and ~22mV for v2. The measured dI/dV spectra and estimated intrinsic v1/v2 peak are displayed in Supplementary Fig. 3, showing a nearly isolated peak.

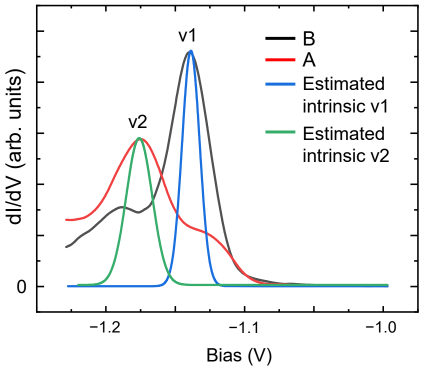

Supplementary Figure 3. dI/dV spectrums at the B/A sites and corresponding estimated intrinsic v1/v2 peak in 54.1° TB-WSe_2.

Supplementary Note 3: dI/dV spectrums near conduction band edges

Besides the sharp peaks we observed at VB edge, ultra-flat bands have also been predicted to emerge near CB edge due to the modulating triangular potential wells in twisted bilayer TMDs for twist angle around 60°[8]. To explore the predicted flat bands besides VB edge, expanded views of electronic states close to CB edge are shown in Supplementary Fig. 4. Examining the spectra from different sites (Supplementary Fig. 4a for 58.4° TB-WSe_2), we notice that A sites take the CB minimum with an energy difference of ~130meV to other sites, which has been further confirmed by dI/dV mapping at 1.1V (Supplementary Fig. 4b-c). As shown in Supplementary Fig. 4d-g, a similar feature has also been found for 57° ~57.8°, while the 54.1° not. Since the VB maximum lies at B sites for a twist angle of 57°-58.4°, the spatial separation of holes and electrons in TB-WSe_2 could lead to long-lifetime confined excitons. Although the spatial localizations of CB minimum agree with prediction, we fail to observe sharp peaks associated with flat bands near CB edges. A possible reason might be the STS method we use to measure LDOS. The constant-height dI/dV spectra we use are sensitive to states around the Γ point but weak to detect other states with large parallel momentum, such as the states near the K point of TMDs[9]. The calculated flat bands near CB edge are folded from the K and K' points of the top and bottom layers, which may explain the absence of sharp peaks near CB in our measurements.

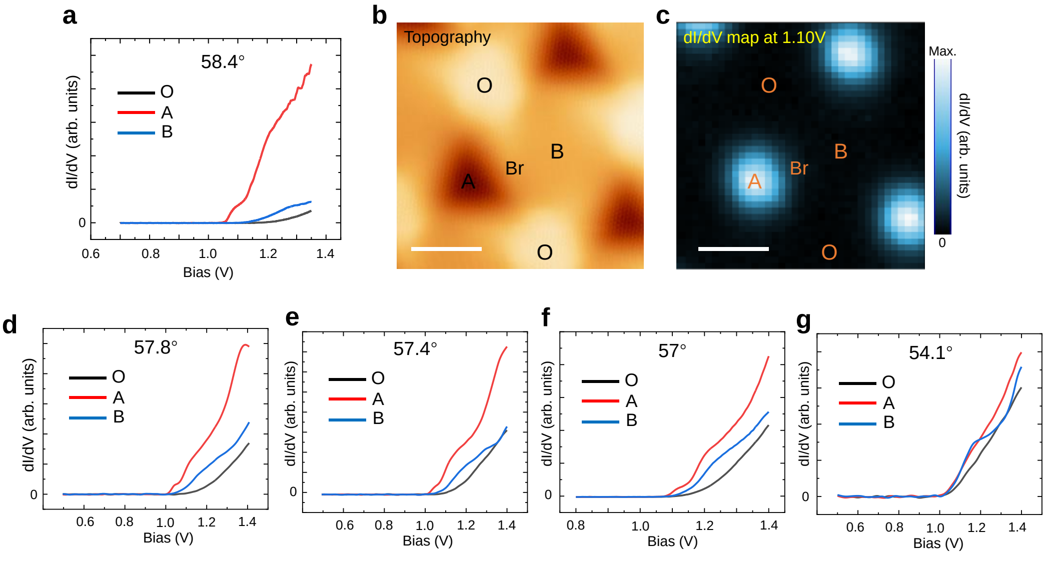

Supplementary Figure 4. Spectroscopy near conduction band edge of 54.1°, 57°, 57.4°,

57.8° and 58.4° TB-WSe_2. (a) dI/dV spectrums (V_{mod}=5mV, 787.3Hz) near CB edge of 58.4° TB-WSe_2, showing that A sites take the CB minimum. (b) An STM topographic image (−1.2V, 1nA) of 58.4° TB-WSe_2. (c) Corresponding dI/dV map of (b) acquired at 1.10 V. Scale bar in (b) (c) is 5nm. (d)-(g) dI/dV spectrums (V_{mod}=15mV, 787.3Hz) near CB edge at three moiré sites for a twist angle of 57.8°, 57.4°, 57°, and 54.1°, respectively.

Supplementary Note 4: Moiré potential at different twist angles

To explicitly show the effects of the in-plane lattice reconstruction, we also calculate the flat band properties of 58.4° TB-WSe_2 using the continuum model with a potential $V_M(\boldsymbol{r}) = 2V_0 \sum_i \cos(\boldsymbol{g_i} \cdot \boldsymbol{r} + \phi)$, similar to the 54.1° case. This moiré potential is produced by the out-of-plane deformation without considering the in-plane lattice reconstruction. Based on a set of parameters $(m^*, V_0, \phi) = (1.2m_e, 40meV, 170°)$, Supplementary Fig. 5 shows the calculated band structure and LDOS maps of the first six flat-band wavefunctions. The E1/E2 flat-band wavefunctions are distributed in region B, however, E3 state mainly lies at region A. For the flat bands at deeper energies (E4-E6), their wave functions are localized at both A and B regions, avoiding the O region. These flat band features, both spatial localization and wavefunction patterns, are inconsistent with the observed multiple B-confined states in Fig. 3**l**.

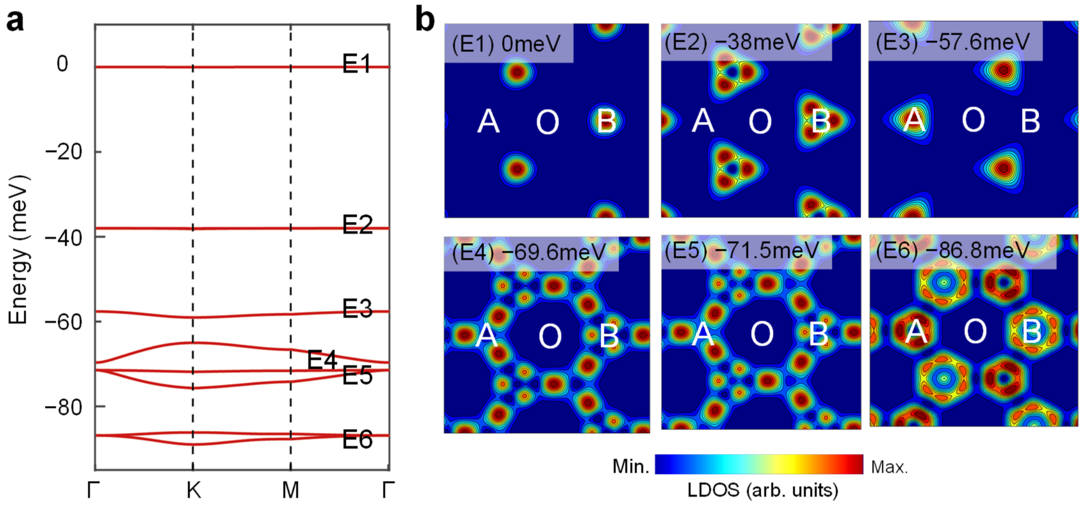

Supplementary Figure 5. Calculated band structure (a) and LDOS maps (b) of 58.4° TB-WSe_2 based on moiré potential $\boldsymbol{V_M(r)}$. Note that the valence band edge is set at E=0.

Concerning spin-orbit coupling (SOC), the energy splitting caused by SOC must be

zero at the Gamma point due to time-reversal symmetry. Slightly away from the Gamma point, the SOC effect is expected to be small. As the moiré Brillouin zone is small (the zone edge to Gamma point distance is about $|\mathrm{k}| \sim \frac{4\pi\theta}{3a} < 0.13\text{Å}^{-1}$), the moiré bands near the valence band maximum originate from bands with small SOC effects. Therefore, the effect of SOC is negligible in our study.

Supplementary Fig. 6a shows the effective moiré potential $V_M(\boldsymbol{r})$ (54.1°) spatially varied with the moiré lattice, originating from inhomogeneity of the interlayer hybridization in the moiré superlattice. As the twist angle varies from 57° to 58.4°, the measured spatial distribution of the states is sequentially consistent for 57°-58.4° TB-WSe_2, following the triangular symmetry. It indicates the triangular potential (V_C) caused by lattice reconstruction gradually involves as the twist angle ranging from 57° to 58.4°. Note that previous DFT calculations show the enhanced confining potential (V_C) as the twist angle close to 60°[8]. Therefore, in reconstructed case, we do this assumption that reconstruction-induced $V_C(\boldsymbol{r})$ gradually plays dominate roles in modulating the electrons while the inhomogeneous hybridization-induced $V_M(\boldsymbol{r})$ becomes relatively insignificant. Supplementary Fig. 6b-e show the moiré potential with twist angle-dependent parameters (V_0, V_1) used in our calculations.

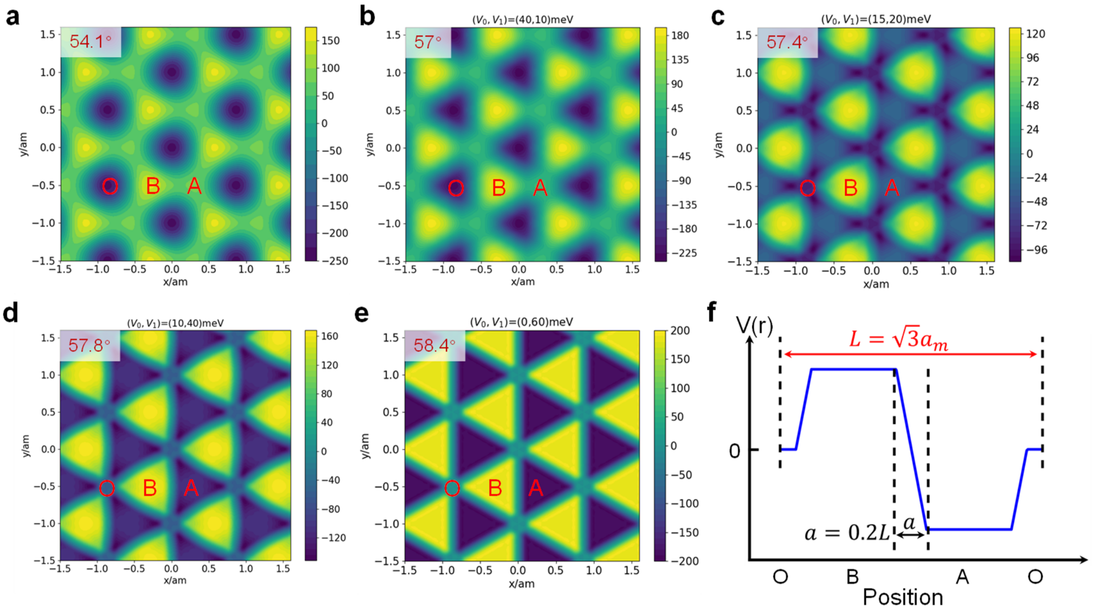

Supplementary Figure 6. The moiré potential profile at different twist angles. (a) $\theta = 54.1°$; (b) $\theta = 57°, (V_0, V_1) = (40, 10)$meV; (c) $\theta = 57.4°: (V_0, V_1) = (15, 20)$meV; (d)

$\theta = 57.8°: (V_0, V_1) = (10, 40)\text{meV}$; (e) $\theta = 58.4°: (V_0, V_1) = (0, 60)\text{meV}$. (f) The parameters we used for the profile of $V_C(r)$. We adopt this form of potential which is close to the previous DFT calculation[8]. It is worth noting that a characterizes the slope of the potential near the boundary of triangular domains, and when $a \to 0$, $V_C(r)$ becomes an ideal triangular well.

Supplementary References:

1. Wilson NR, *et al.* Determination of band offsets, hybridization, and exciton binding in 2D semiconductor heterostructures. *Sci. Adv.* **3**, e1601832 (2017).
2. Bussolotti F, Kawai H, Wong SL, Goh KEJ. Protected hole valley states in single-layer MoS_2. *Phys Rev B* **99**, 045134 (2019).
3. Li H, *et al.* Imaging moiré flat bands in three-dimensional reconstructed WSe_2/WS_2 superlattices. *Nat. Mater.* **20**, 945-950 (2021).
4. Ugeda MM, *et al.* Giant bandgap renormalization and excitonic effects in a monolayer transition metal dichalcogenide semiconductor. *Nat. Mater.* **13**, 1091-1095 (2014).
5. Zhang Z, *et al.* Flat bands in twisted bilayer transition metal dichalcogenides. *Nat. Phys.* **16**, 1093-1096 (2020).
6. Feenstra RM, Stroscio JA. Tunneling spectroscopy of the GaAs (110) surface. *J. Vac. Sci. Technol. B* **5**, 923–929 (1987).
7. Machida T, Kohsaka Y, Hanaguri T. A scanning tunneling microscope for spectroscopic imaging below 90 mK in magnetic fields up to 17.5 T. *Rev. Sci. Instrum.* **89**, 093707 (2018).
8. Naik MH, Kundu S, Maity I, Jain M. Origin and evolution of ultraflat bands in twisted bilayer transition metal dichalcogenides: Realization of triangular quantum dots. *Phys. Rev. B* **102**, 075413 (2020).
9. Zhang C, *et al.* Probing Critical Point Energies of Transition Metal Dichalcogenides: Surprising Indirect Gap of Single Layer WSe_2. *Nano Lett* **15**, 6494-6500 (2015).
